# Digital health applications, telemedicine and large language models in dermatology: results of a Germany‐wide survey

**DOI:** 10.1111/ddg.70019x

**Published:** 2026-01-19

**Authors:** Lukas Henk, Leah Schirren, Felix Mühlensiepen, Claudia Traidl‐Hoffmann, Marina Otten, Victor Olsavszky, Alexander Zink, Michael Hertl, Sebastian Kuhn, Johannes Knitza

**Affiliations:** ^1^ Institute for Digital Medicine School of Medicine Philipps‐Universität Marburg Marburg Germany; ^2^ Department of Dermatology and Allergology School of Medicine Philipps‐Universität Marburg Marburg Germany; ^3^ Faculty of Health Sciences Brandenburg Medical School Theodor Fontane Neuruppin Germany; ^4^ Institute of Environmental Medicine and Integrative Health Medical Faculty University of Augsburg Augsburg Germany; ^5^ CK‐CARE, Christine Kühne, Center for Allergy and Research and Education Davos Switzerland; ^6^ Institute for Health Services Research in Dermatology and Nursing University Medical Center Hamburg‐Eppendorf Hamburg Germany; ^7^ Department of Dermatology, Venereology and Allergology, University Medical Center and Medical Faculty Mannheim University of Heidelberg and Center of Excellence in Dermatology Mannheim Germany; ^8^ Department of Dermatology and Allergy Biederstein School of Medicine Technical University of Munich Munich Germany

**Keywords:** ChatGPT, eHealth, large language models, mobile applications, Telemedicine

## Abstract

**Background**: Digital technologies offer opportunities for dermatological care, but there is a lack of systematic data on their use and acceptance, particularly with regard to digital health applications (DiGA) and large language models (LLMs).

**Patients and Methods**: From November 2024 to January 2025, an anonymous online survey was conducted among German dermatologists as part of continuing education courses. The survey covered the use of and attitudes towards telemedicine, DiGA and LLMs, as well as competence assessments and education needs.

**Results**: 100 dermatologists participated (64% female, average age 40.6 years). 58% stated that they did not currently offer telemedicine services; only 5% regularly used video consultations. 21% had already prescribed DiGA, with the majority (78%) seeing this as the responsibility of general practitioners. 96% of dermatologists believed that DiGA could improve medical care. The main barriers cited were lack of knowledge (76%), lack of time (74%), insufficient remuneration and a complex prescription process (43% each). 45% of respondents had used LLMs in a professional context. 76% rated the impact of digitalization on patient care as positive, and 80% expressed a desire for training opportunities on the use of digital technologies in everyday medical practice.

**Conclusion**: Digitalization and, in particular, DiGA are viewed positively by most dermatologists, but digital services are hardly used at present. Targeted knowledge expansion and specific training opportunities could significantly promote the implementation of digital technologies in everyday dermatological practice.

## INTRODUCTION

Digital technologies are increasingly shaping medical care and offer numerous opportunities to optimize diagnostics and therapy, particularly in dermatology.^1^, ^2^ With the publication of the first S2k guideline on teledermatology in 2020, dermatology was the first medical specialty in Germany to systematically address telemedical care models within the framework of an evidence‐based guideline.^3^ In addition, in 2023 the German Dermatological Society (DDG) and the Professional Association of German Dermatologists (BVDD) formulated perspectives on the further development of digital care structures in dermatology in a joint position paper, emphasizing in particular the integration of health apps and artificial intelligence as well as the need for specific medical training offers.^4^

A cross‐sectional survey conducted in 2019 and 2021 documented an increase in teledermatology video consultations during the COVID‐19 pandemic.^5^ Currently, 69 digital health applications (DiGA) are listed (as of July 2025), none of which have yet been explicitly approved for dermatological indications. However, some applications address relevant comorbidities such as depression, smoking cessation, arterial hypertension, or obesity.^6^, ^7^ DiGA are reimbursed by all statutory health insurers and by many private health insurers. The comprehensive review of safety and efficacy by the Federal Institute for Drugs and Medical Devices (BfArM) offers patients and physicians a decisive advantage over the otherwise often rapidly changing and non‐transparent app offerings. In parallel, large language models (LLMs) such as ChatGPT are becoming increasingly important. Potential applications range from supporting medical history‐taking, generating discharge letters and patient information, to the structured evaluation of guidelines.^2^ The extent to which dermatologists currently use video consultations, DiGA and LLMs is still unclear.

The aim of this study was therefore to systematically assess the use, acceptance and perceived barriers of digital technologies in dermatology. The focus was on DiGA and LLMs. In addition, the need for training in the use of digital technologies among dermatologists was assessed.

## METHODS

Data collection was carried out digitally using an anonymous online questionnaire (Google Forms). The results were reported in accordance with the CHERRIES recommendations (Checklist for Reporting Results of Internet E‐Surveys).^8^ Dermatologists were recruited by study staff at dermatology training events (Derma Update Congress, Berlin, as well as local training courses at the University Hospital Marburg) between November 2024 and January 2025. Participants could either complete the questionnaire directly on a provided tablet or via a QR‐code on their own device. Participation was voluntary and without financial or material compensation. Only fully completed questionnaires were included in the analysis. The Ethics Committee of Philipps University Marburg confirmed that no approval was required for this anonymous survey (24‐292 ANZ).

The questionnaire consisted of 16 questions (online supplement S1). In addition to sociodemographic characteristics, the use of telemedical applications and LLMs was surveyed. Also collected were self‐assessment of digital skills, individual training needs, and perceptions of the impact of digitalization on patient care. A particular focus was on DiGA, with six questions covering use, acceptance and perceived barriers.

For data analysis, Microsoft Excel for Mac (Microsoft Corporation, Redmond, WA, USA) and IBM SPSS Statistics, Version 30.0.0.0 (IBM Corporation, Armonk, NY, USA) were used. Associations between ordinal variables were analyzed using Spearman's rank correlation (Spearman rho). Results are presented in the text as ρ = [correlation coefficient], p = [p‐value].

## RESULTS

### Demographics

A total of 100 participants completed the questionnaire, of whom 64% identified as female and 36% as male. The average age was 40.6 years. Most participants worked at university hospitals (44%), followed by solo practices (28%) and group practices (18%) (Table 1).

**TABLE 1:** Demographics.

**Characteristics**	**Respondents**
**Gender, n (%)**	
Female	64 (64)
Male	36 (36)
Diverse	0
**Age (years**)**, n (%)**	
20‐30	23 (23)
31‐40	34 (34)
41‐50	22 (22)
51‐60	13 (13)
>61	8 (8)
**Occupational position, n (%)**	
Single practice	28 (28)
Group practice	18 (18)
Medical care center	4 (4)
General care hospital	1 (1)
Tertiary care hospital	5 (5)
University hospital	44 (44)

### Use of telemedicine

Regarding the proportion of patients treated via telemedicine, 58% reported not currently offering any telemedical services. A further 27% used teledermatology for less than 10% of their patients. Only 12% reported treating a higher proportion (10% to 50%) via telemedicine. The most frequently used telemedical applications were asynchronous communication between colleagues (70% “often” or “rather often”), synchronous communication between colleagues (54% “often” or “rather often”), and asynchronous communication with patients (54% “often” or “rather often”). Video consultations were used “often” or “rather often” by only 5% of respondents (Figure 1). Asynchronous communication referred to, for example, consultation requests or emails, while synchronous communication primarily meant phone calls or video conferences.

**FIGURE 1:**
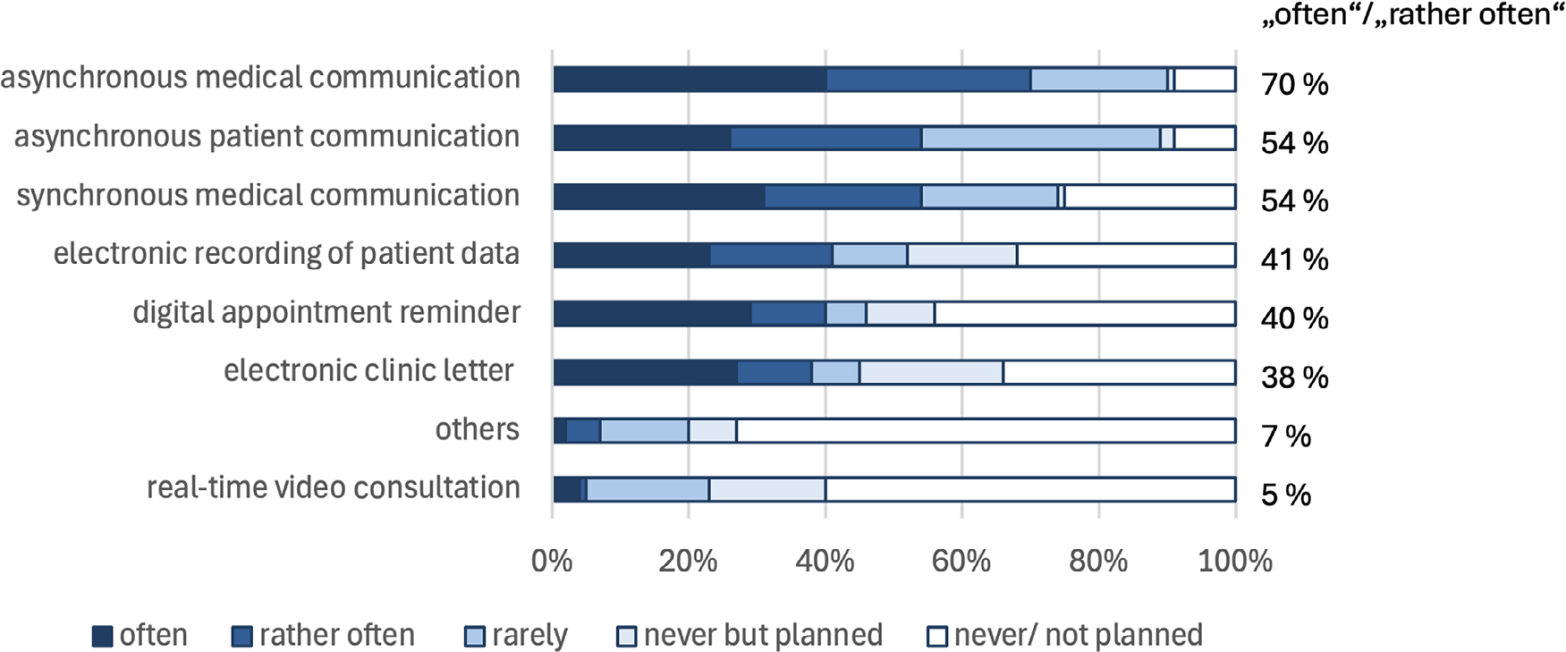
Use of digital tools in routine clinical practice.

### Acceptance, use and barriers regarding DiGA

96% of dermatologists surveyed saw great potential in DiGA to improve care. Although no DiGA are currently approved for dermatological indications, 97% saw potential in dermatology‐specific DiGA. 21% reported having prescribed a DiGA at least once. Of these, 14% prescribed a DiGA about once a month, 2% about weekly and 5% almost daily. Responsibility for prescribing was seen primarily with general practitioners (78%), followed by other specialists (63%) (Figure 2). Most participants rated their own knowledge of digital health applications as rather low (4/10; IQR: 3). Limited knowledge was also the most frequently cited barrier to prescribing (76%), followed by lack of time for counseling and instruction (74%), inadequate reimbursement, and a perceived complicated prescribing process (each 43%) (Figure 3).

**FIGURE 2:**
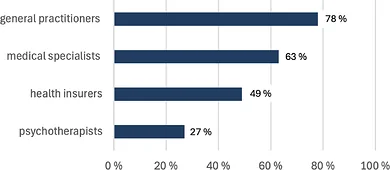
Responsibility for prescribing and providing information about DiGA from the perspective of respondents (multiple answers possible).

**FIGURE 3:**
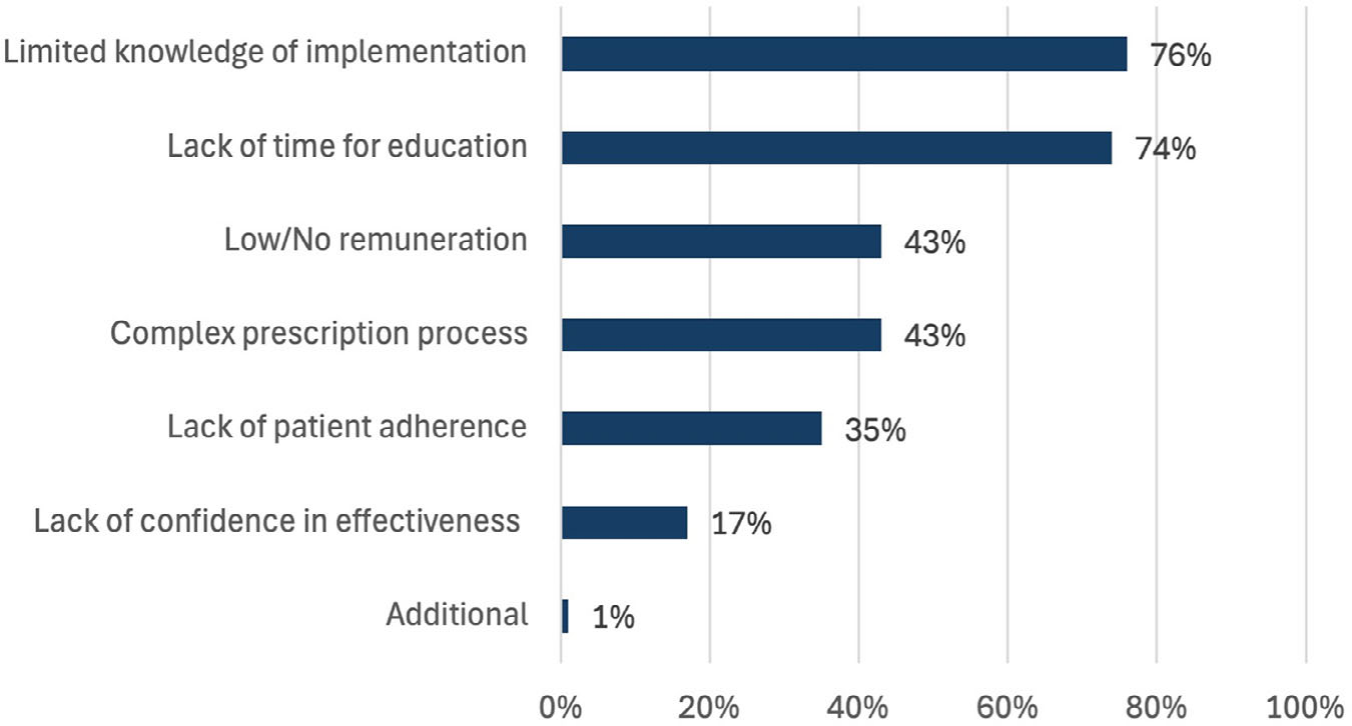
Perceived barriers to prescribing DiGA (multiple selections possible).

### Use of large language models

45% of dermatologists reported using LLMs in their professional context, 25% monthly, 14% weekly and 6% daily (Figure 4). Correlation analysis showed a moderate, significant negative correlation between participant age and use in the professional context (ρ = ‐0.417, p < 0.001).

**FIGURE 4:**
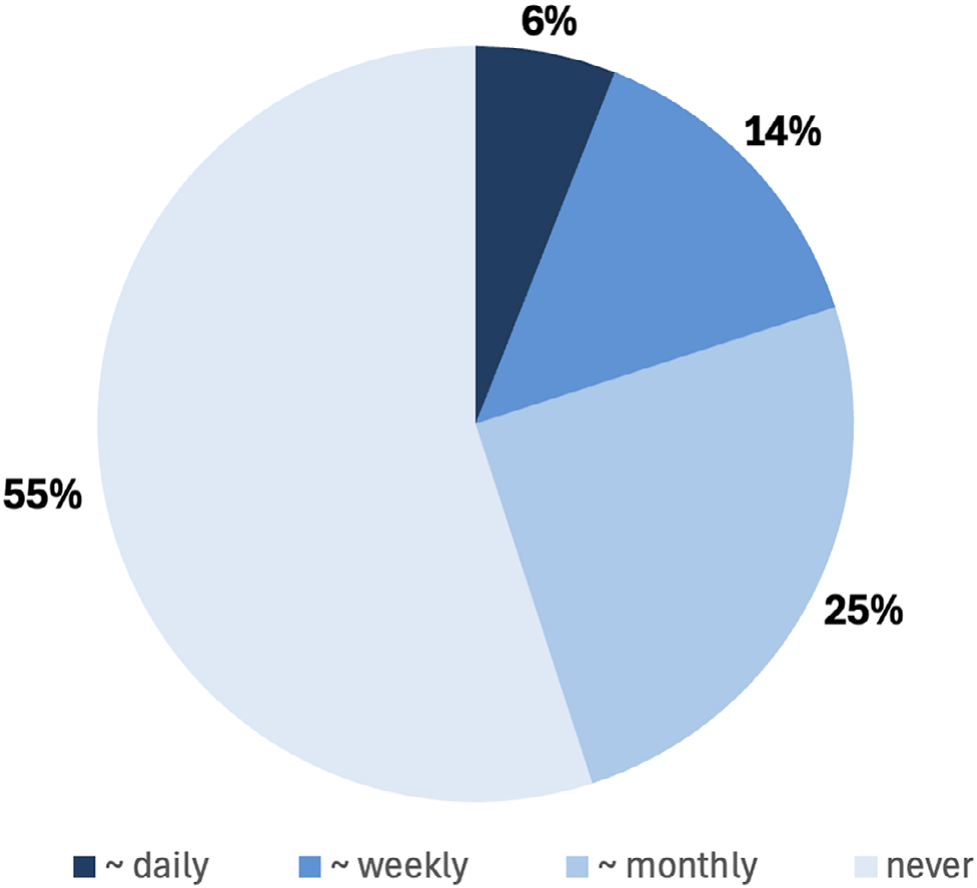
Use of large language models in the context of medical practice.

### Attitudes, self‐assessed competencies and training needs

76% of respondents rated the impact of digitalization on patient care as (very) positive, only 1% as (very) negative, the rest gave a neutral rating. 42% rated their knowledge of digital medicine as good, while 23% rated their knowledge as insufficient. With regard to confidence in handling medical digital and online applications, 56% rated it as good, while 12% felt insecure. A Pearson correlation analysis showed no significant association between age and self‐assessed knowledge in digital medicine (ρ = 0.046, p = 0.65) or perceived confidence in using medical digital applications (ρ = 0.011, *p* = 0.91). 80% of respondents would welcome training or continuing education on the use of digital tools in clinical practice.

## DISCUSSION

The results of this study show that digital technologies, especially DiGA, are generally viewed positively by dermatologists. Nevertheless, the actual use of telemedical procedures remains limited. The low prescribing rate of DiGA and the low use of video consultations highlight the discrepancy between theoretical acceptance and practical application. Compared to earlier surveys documenting increased use of video consultations during the COVID‐19 pandemic, our data again show a decline.^5^ This trend was also confirmed in a 2023 survey of physicians treating patients with chronic urticaria.^9^

A central finding was the low prescription rate of DiGA, although 96% of participants acknowledged their potential benefit for care. 21% reported having prescribed a DiGA at least once, a figure that is clearly below prescribing rates in other specialties such as general practice or internal medicine.^10^, ^11^ The low prescribing frequency may be mainly due to the lack of approved DiGA for dermatological indications. At present, use in dermatology is limited to DiGA for comorbidities. Especially in chronic conditions such as allergies, psoriasis or hidradenitis suppurativa, where lifestyle factors and active self‐management play a central role, DiGA could make an important contribution.^12^, ^13^, ^14^, ^15^

A major barrier was the perceived lack of knowledge about digital health applications, which was also the most frequently cited obstacle to prescribing. These findings are consistent with studies from other specialties, where lack of information, limited time resources and unclear reimbursement structures were identified as key barriers.^11^, ^16^, ^17^

Although the integration of LLMs into medical care is still in its early stages, this survey shows that they are already relatively frequently used by dermatologists in everyday practice. Future studies should examine in which specific applications LLMs are being used and what potentials and challenges arise in dermatological practice. Notably, there was a significant negative correlation between age and frequency of use. Targeted training could help ensure that these technologies are also used more frequently and appropriately by older colleagues.

The fact that 80% of respondents expressed a desire for training in digital medicine highlights the existing need for qualification. Content on digital medicine is currently insufficiently represented in the model specialty training regulations. Professional societies such as DDG and BVDD have already formulated concrete demands in a joint position paper, emphasizing that physician qualification in the use of digital methods “must be integrated into training, continuing and advanced education to a far greater extent than before”. This demand is reflected, among other things, in the “Teledermatology” certificate of the German Academy of Dermatology (DDA), which specifically trains physicians for telemedical consultations. Additional training offerings on DiGA and LLMs could represent a useful extension here. Scalable online formats, such as the webinars of Digitale Dermatologie e. V., already offer low‐threshold access to digital training. A qualitative study with dermatologists, nurses and patients in dermatology identified low digital health literacy among patients as a central barrier to digital transformation in dermatological care.^18^ This also underscores the need for training on the patient side.

This survey was the first to systematically examine the use of DiGA and LLMs in dermatology. The study has several limitations that should be considered when interpreting the results. The main limitation is the relatively small sample size of 100 participants, which is not representative of all dermatologists. In addition, the surveyed group was relatively young. The fact that younger participants in particular rated their knowledge as rather low and saw a high need for training highlights the relevance. Previous studies have shown that younger professionals use digital tools much more frequently in clinical practice.^19^ The survey was also conducted in the context of voluntary training events, which may have led to a bias due to overrepresentation of young, digitally inclined or training‐oriented dermatologists.

## CONCLUSION

Despite an overall positive attitude toward digital technologies, especially DiGA, their use in dermatological practice remains limited. The survey results revealed not only a clear need for training but also structural and organizational barriers to broad implementation in everyday care. To realize the potential of digital applications for improving dermatological care, targeted training, dermatology‐specific DiGA, appropriate reimbursement models and practical implementation support are needed.

## FUNDING

The study was funded by internal funds of the Institute for Digital Medicine.

## CONFLICTS OF INTEREST STATEMENT

None.

## Supporting information



Supporting Information
