# Supplementary material for: Digital health applications, telemedicine and large language models in dermatology: results of a Germany‐wide survey
Source: J Dtsch Dermatol Ges. 2026 Jan 19;24(10):e1435–9. doi: 10.1111/ddg.70019x (PMC13636116; doi:10.1111/ddg.70019x)
Supplement: Supplementary file 1 [file DDG-24-e1435-s001.pdf]

Fragebogen zur Digitalisierung in der Dermatologie

Vielen herzlichen Dank für Ihre Teilnahme!!!

* Indicates required question

1. Welchem Geschlecht fühlen Sie sich zugehörig? *

Tick all that apply.

- [ ] Weiblich
- [ ] Männlich
- [ ] Divers

2. Wie alt sind Sie? (Jahre in Zahlen) *

____

3. In welcher Position arbeiten Sie? *

Tick all that apply.

- [ ] Einzelpraxis
- [ ] Gemeinschaftspraxis
- [ ] MVZ
- [ ] Klinikum der Grund- und Regelversorgung
- [ ] Klinikum der Maximalversorgung
- [ ] Universitätsklinikum

4. Ich habe gute Kenntnisse im Bereich der digitalen Medizin *

Tick all that apply.

- [ ] Stimme voll und ganz zu
- [ ] Stimme zu
- [ ] Neutral
- [ ] Stimme nicht zu
- [ ] Stimme überhaupt nicht zu

5. Ich fühle mich sicher bei der Nutzung von medizinischen digitalen-/ Onlineanwendungen *

Tick all that apply.

- [ ] Stimme voll und ganz zu
- [ ] Stimme zu
- [ ] Neutral
- [ ] Stimme nicht zu
- [ ] Stimme überhaupt nicht zu

6. Wie groß ist der Anteil der Patienten die Sie telemedizinisch behandeln? (0-100%) *

____

7. Wie häufig verwenden Sie im Rahmen Ihrer ärztlichen Tätigkeit Large Language Models (wie z.B. Chat GPT oder Gemini) *

Tick all that apply.

- [ ] Nie
- [ ] Ca. monatlich
- [ ] Ca. wöchentlich
- [ ] Ca. täglich

8. Wie oft verordnen Sie in Ihrer ärztlichen Tätigkeit digitale Gesundheitsanwendungen? *

Tick all that apply.

- [ ] Nie
- [ ] Ca. monatlich
- [ ] Ca. wöchentlich
- [ ] Ca. täglich

9. Wer ist primär aus Ihrer Sicht für die Verschreibung und Information über digitale Gesundheitsanwendungen (DiGAs) zuständig (Mehrfachantwort)? *

Tick all that apply.

- [ ] Hausärzt*innen
- [ ] Fachärzt*innen
- [ ] Psychotherapeut*innen
- [ ] Krankenkassen

10. Wie gut schätzen Sie Ihr Wissen zu digitalen Gesundheitsanwendungen (DiGAs) ein? *

Mark only one oval.

	1	2	3	4	5	6	7	8	9	10	
kein	◯	◯	◯	◯	◯	◯	◯	◯	◯	◯	sehr großes Wissen

11. Denken Sie, digitale Gesundheitsanwendungen können die Versorgung verbessern? *

Tick all that apply.

- [ ] ja
- [ ] nein

12. Denken Sie dermatologische digitale Gesundheitsanwendungen können die Versorgung verbessern (z.B. psychologische Unterstützung für SLE, Psoriasis Patienten)? *

Tick all that apply.

- [ ] ja
- [ ] nein

13. Was sehen Sie als die größte Barriere bei der Verschreibung von DiGAs? (Mehrfachauswahl) *

Tick all that apply.

- [ ] geringe/ kein Vergütung
- [ ] geringes Wissen über Implementierung
- [ ] Mangelndes Vertrauen in Wirksamkeit
- [ ] Komplizierter Verschreibungsprozess
- [ ] Mangelnde Adhärenz von Patient*innen
- [ ] Mangelnde Zeit für Aufklärung/ Einweisung
- [ ] Weitere

14. Welche Verfahren der digitalen Medizin setzen Sie in Ihrer Praxis ein *

Mark only one oval per row.

	oft	eher oft	selten	nie, aber geplant	nie, nicht geplant
**Echtzeit Videosprechstunde**	◯	◯	◯	◯	◯
**Asynchrone Patientenkommunikation (z.B. Email, SMS Textnachrichten)**	◯	◯	◯	◯	◯
**Echtzeit Kommunikation für fachlichen Austausch**	◯	◯	◯	◯	◯
**Asynchrone Kommunikation für fachlichen Austausch (z.B. Email, SMS, Textnachrichten)**	◯	◯	◯	◯	◯
**Elektronische Erinnerung an Termine für Patientin**	◯	◯	◯	◯	◯
**Elektronische Arztbriefe**	◯	◯	◯	◯	◯
**Elektronische oder webbasierte Erfassung von Patientendaten und Bildbefunden (z. B. Fragebögen, Bilder, Wearables und Körperwerte)**	◯	◯	◯	◯	◯
**Andere**	◯	◯	◯	◯	◯

15. Wie schätzen Sie den Einfluss der Digitalisierung auf die Patientenversorgung ein? *

Mark only one oval.

	1	2	3	4	5	
sehr	◯	◯	◯	◯	◯	sehr positiv

16. Würden Sie sich Schulungen/Fortbildungen wünschen zum Einsatz von digitalen Tools in der klinischen Praxis? *

Tick all that apply.

- [ ] ja
- [ ] nein
- [ ] weiß nicht

This content is neither created nor endorsed by Google.

Google Forms
